# Technological Aspects of Lithium-Titanium Ferrite Synthesis by Electron-Beam Heating

**DOI:** 10.3390/ma16020604

**Published:** 2023-01-08

**Authors:** Elena Lysenko, Vitaly Vlasov, Evgeniy Nikolaev, Anatoliy Surzhikov, Sergei Ghyngazov

**Affiliations:** Problem Research Laboratory of Electronics of Dielectrics and Semiconductors, Research School of Physics, Tomsk Polytechnic University, Lenina Avenue 30, 634050 Tomsk, Russia

**Keywords:** lithium-titanium ferrite, X-ray diffraction analysis, mechanical activation, radiation-thermal heating, electron beam

## Abstract

Solid-phase synthesis of lithium-titanium ferrite by electron-beam heating of a Fe_2_O_3_–Li_2_CO_3_–TiO_2_ initial reagents mixture with different history (powder, compact, mechanically activated mixture) was studied using X-ray diffraction, thermomagnetometric and specific saturation magnetization analyses. Ferrite was synthesized using an ILU-6 pulsed electron accelerator; it generated electrons with electron energy of 2.4 MeV to heat samples to temperatures of 600 and 750 °C. The isothermal holding time upon reaching the synthesis temperature was 0–120 min. The efficiency of ferrite synthesis by electron-beam heating was evaluated via comparison of the characteristics of the obtained samples with those synthesized by conventional ceramic technology under similar temperature-time conditions. It was found that the rate of ferrite formation depends on the heating method, temperature, synthesis time, density, and activity of the initial mixture. It was shown that sample compaction provides the preferential formation of unsubstituted lithium ferrite of Li_0.5_Fe_2.5_O_4_ composition with a Curie temperature of at ca. 630 °C in both synthesis methods. High-energy electron-beam heating of the mechanically activated mixture significantly accelerates synthesis of Li_0.6_Fe_2.2_Ti_0.2_O_4_ substituted ferrite, for which the Curie temperature and specific saturation magnetization were recorded as 534 °C and 50 emu/g, respectively. Therefore, LiTi ferrites can be obtained at a lower temperature (750 °C) and with a shorter synthesis time (120 min) compared to traditional ceramic technology.

## 1. Introduction

Ferrites hold a specific place among the many technological magnetic materials. Ferrite materials, including lithium-containing ferrites, are currently the key elements in most modern radio engineering, electronic, and computing devices [1,2,3,4]. Lithium and substituted lithium ferrites are characterized by a sufficiently low cost, yet they exhibit a certain combination of electrical and magnetic properties, which enable their use in microwave technology. They show high values of the Curie temperature and saturation magnetization, and low dielectric and magnetic losses required to design ferrite devices operating at microwave and millimeter wave frequencies [5,6,7,8].

Lithium-containing ferrites used in microwave technology are typically of a complex composition, which is attained by replacing iron ions, for example, with titanium, zinc, or manganese ions. The electrical properties of ferrites, in particular, can be improved via Ti^4+^ ions introduced into pure lithium ferrite Li_0.5_Fe_2.5_O_4_ to decrease the formation of Fe^3+^ ions during ferrite synthesis [9,10,11]. Therefore, the Li_0.5(1+x)_Fe_2.5−1.5x_Ti_x_O_4_ substituted lithium-titanium ferrite with a spinel structure and high electrical resistance is formed. Substitution with titanium ions increases the electrical resistance, yet the saturation magnetization decreases slightly. The Curie temperature also decreases depending on the concentration of introduced titanium ions.

Several methods have been developed for ferrite synthesis with regard to the field of their application. These include a widespread ceramic technology [12,13,14,15], sol-gel [16,17], co-precipitation method [18], etc. [19,20]. The above methods are aimed at synthesis of ferrites with specified, reproducible, and uniform electromagnetic properties.

The conventional ceramic technology, the key method for ferrite synthesis, employs solid-phase synthesis of ferrites from mixtures of initial oxides that include iron oxide Fe_2_O_3_ as the main component. To obtain ferrites of complex compositions, ceramic technology requires high synthesis temperatures and long high temperature exposures to synthesize ferrites with good properties [13,14].

It is known that the properties of ferrites synthesized by this method significantly depend on the redox processes occurring during heating and cooling. In this case, high temperatures stimulate reduction reactions associated with oxygen loss from the ferrite lattice [21]. Volatile elements, such as lithium, in ferrite chemical composition may complicate the processes. As reported in [21], lithium in combination with oxygen can be released from samples at 1000 °C. Therefore, in many cases, high temperatures used for synthesis of lithium-containing ferrites become critical due to the violation of their stoichiometric composition and, as a result, deterioration of the synthesized sample properties.

This problem can be partially solved by mechanical milling of the initial reagents in a ball mill [22,23,24,25,26,27,28,29,30,31]. Such treatment reduces the size of the powder particles and simultaneously increases imperfection of the powders, which enhances the reactive activity of the reagents. As shown in [22,32], grinding of the initial oxides, used for ferrite formation, in a planetary mill reduces the synthesis temperature by 100–200 °C, depending on ferrite composition. The time of mechanical activation of the powder typically ranges from 1 to 5 h, depending on the energy intensity of the treatment, which depends on the rotation speed of the mill cups, the diameter of the balls, etc.

A synthesis method was previously developed to reduce the temperature and time of ferrite synthesis; it employs heating of the initial reagents by an electron beam with energies above 1 MeV [33,34,35,36,37,38,39,40,41]. High electron energies provide the possibility of volumetric heating of the synthesized samples, and the electron penetration depth depends on the density of the samples. The synthesis temperature can be controlled by changing the parameters of the electron beam, including the current density.

The electron-beam heating method was used to synthesize ferrites of various compositions, including spinel lithium, lithium-zinc and manganese-zinc ferrites, and hexagonal ferrites [35,38,39,40,41,42]. In [43,44,45], it was shown that this heating method increases the rate of solid-phase interaction between the initial reagents, which significantly decreases the ferrite synthesis temperature. The results reported in [43] showed that the initial reagents mainly interact during nonisothermal heating of the samples within a very short time. Formal kinetic analysis of lithium ferrite synthesis showed that ferrite formation under electron-beam heating is accelerated due to the decreased activation energy of synthesis and the decreased pre-exponential factor in the temperature dependence of the reaction rate.

To develop the electron beam technology for ferrite production, the efficiency of ferrite synthesis under these conditions should be increased, which depends on numerous parameters, including treatment modes and sample history. This study focused on solid-phase synthesis of lithium-titanium ferrite by electron-beam heating of samples of different density and activity.

## 2. Materials and Methods

We studied synthesis of ferrite with the chemical formula Li_0.6_Fe_2.2_Ti_0.2_O_4_, which was fabricated from the commercially initial reagents Li_2_CO_3_ (10.38 wt.%), Fe_2_O_3_ (82.14 wt.%), and TiO_2_ (7.48 wt.%) (powder purity 99%, Reahim Co., Moscow, Russia). The average particle sizes of Li_2_CO_3_, Fe_2_O_3_, and TiO_2_ powders were 47, 12, and 1 μm, respectively.

The mixture of Li_2_CO_3_–Fe_2_O_3_–TiO_2_ initial reagents was divided into several parts to obtain samples of different density and activity. Samples in the form of reagent powder showed a bulk density of 1.7 g/cm^3^. Samples in the form of pellets were compacted using a hydraulic press under pressure of 200 MPa; the pellet density was 2.8 g/cm^3^.

Some of the samples made from the initial powder mixture were mechanically activated in an AGO-2S planetary ball mill at a rotation speed of 1290 (MA1 samples) and 2220 (MA2 samples) rpm. For milling, steel cups and 6 mm balls were used. The ball-to-powder mass ratio was 10:1.

Ferrite synthesis was performed by two methods. The first method was a conventional ceramic technology; samples were synthesized by thermal heating (T-samples) in the laboratory furnace at 600 and 750 °C with an isothermal holding time of up to 120 min. The second method employed radiation-thermal heating of samples (RT-samples); synthesis was performed by high-energy electron-beam heating using an ILU-6 pulsed electron accelerator with electron energy of 2.4 MeV. The time of the nonisothermal heating stage varied from 3 to 5 min with respect to the synthesis temperature, which depended on the beam current density. More details about the RT method of sample treatment are reported in [45].

An ARL X’TRA X-ray diffractometer, the Powder Cell 2.5 software, and the PDF-4+ powder database were used for X-ray diffraction analysis (XRD) of the samples. The specific saturation magnetization (σ_S_) was measured using an H-04 induction magnetometer at a magnetizing field of 4.7 kOe. The Curie temperature (*T_C_*) of the samples was studied by the thermomagnetometric method based on thermogravimetric measurements with an applied external magnetic field. The temperature was analyzed using a Netzsch STA 449C Jupiter thermal analyzer and permanent magnets providing an external magnetic field of 5 Oe. The thermomagnetometry technique is described in more detail in [46].

## 3. Results and Discussion

### 3.1. XRD Analysis

Formation of lithium-titanium ferrite proceeds in accordance with Equation (1):

Li_2_CO_3_ + (5 − 3x)(x + 1)^−1^Fe_2_O_3_ + 4x(x + 1)^−1^TiO_2_→4(x + 1)^−1^Li_0.5(1+x)_Fe_2.5−1.5x_Ti_x_O_4_ + CO_2_ (1)

During synthesis, at x = 0.2, soft magnetic spinel ferrite of the Li_0.6_Fe_2.2_Ti_0.2_O_4_ composition, is formed. In [44], it was shown that lithium-titanium ferrite synthesis can yield lithium ferrite in accordance with the reaction:

Li_2_CO_3_ + 5Fe_2_O_3_ → 4Li_0.5_Fe_2.5_O_4_ + CO_2_ (2)

and substituted spinel phases of lithium-titanium ferrite with *x* ≠ 0.2. These spinel phases are typically transition products of the reaction; their concentration depends on the temperature-time treatment conditions. At a sufficiently high synthesis temperature and long isothermal exposure, the transition products interact with each other to form Li_0.6_Fe_2.2_Ti_0.2_O_4_ ferrite.

In [44], it was shown that the lattice parameters of the Li_0.5_Fe_2.5_O_4_ and Li_0.5(1+x)_Fe_2.5−1.5x_Ti_x_O_4_ ferrite phases are similar, which causes the merge of the reflections in the diffraction patterns. Therefore, the total content of spinel ferrite phases formed during ferrite synthesis was analyzed by the XRD method. 

**Figure 1:**
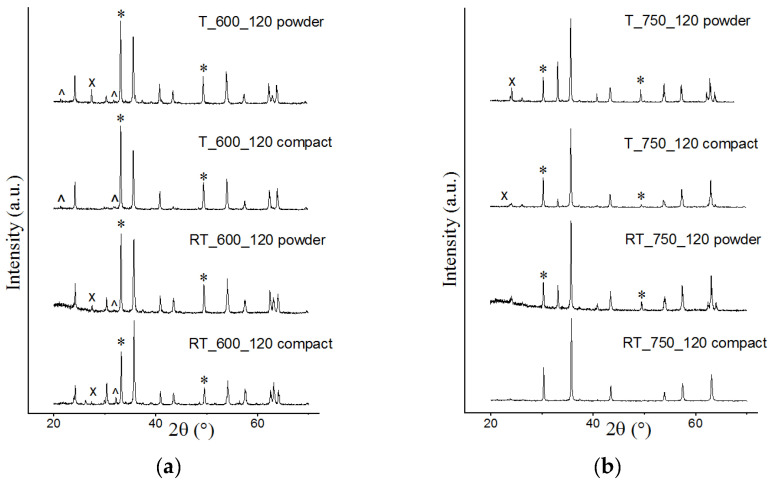
X-ray diffraction patterns of LiTi ferrite synthesized at 600 (**a**) and 750 (**b**) °C by T and RT heating of powdered and compacted Li_2_CO_3_/Fe_2_O_3_/TiO_2_ mixture. *—Fe2O3, ^—Li2CO3, ^X^—TiO_2_.

The XRD patterns were obtained for all the studied samples. The paper reports data for samples synthesized for 120 min. Figure 1 shows the XRD patterns of samples synthesized from powdered and compacted mixtures of initial reagents. The analysis showed that after synthesis the samples contained not only spinel phases, which may belong to lithium and titanium-substituted lithium ferrites, but also phases of unreacted initial components, mainly Fe_2_O_3_ (marked reflections), which quantitative content depends on synthesis conditions. Consequently, the amount of the synthesized spinel ferrite phase grows, and the amount of powder starting reagents decreases at increased temperature and isothermal holding time in both synthesis methods.

Table 1 and Table 2 present XRD and other data for the spinel phase synthesized within 120 min in different samples. The lattice parameter for this phase synthesized in powdered and compacted samples is about 8.33 Ǻ. Lithium ferrite of the Li_0.5_Fe_2.5_O_4_ composition [47] exhibits this value of the lattice parameter. It is known that at increased titanium content in the spinel phase, the lattice parameter increases and amounts to 8.335 Ǻ for Li_0.6_Fe_2.2_Ti_0.2_O_4_ ferrite [47]. Therefore, the values obtained for the lattice parameter of the spinel phase in mechanically activated samples most likely evidence the formation of titanium-substituted lithium ferrites.

It was found that the concentration of the spinel phase of ferrite was higher in compacted samples compared to the powder synthesized under similar temperature-time regime by both heating methods. This phenomenon was interpreted in our study [32], which shows that the decomposition of lithium carbonate and its interaction with iron oxide can be accelerated at an increased compact density of mixtures of Fe_2_O_3_—Li_2_CO_3_ powder reagents. This accelerates the synthesis process as a whole and intensifies the formation of the ferrite phase. A similar effect can be observed in a synthesis of ferrite from mechanically activated powder, only with a much greater efficiency due to a smaller particle size, high inter-particle contact density, and additional imperfection induced by powder activation.

The analysis also showed that electron-beam heating significantly accelerates the formation of spinel phases in comparison with thermal heating. At a synthesis temperature of 600 °C, the samples contain a large proportion of initial reagents in addition to the formed spinel lithium-titanium ferrite phases. However, the concentration of the initial reagents in the PT samples is significantly lower than that in the samples synthesized by thermal heating. As temperature increased up to 750 °C, formation of lithium-titanium ferrite phases accelerates. As a result, the diffraction patterns for samples synthesized from compacted reagents at 750 °C for 120 min show the content of only the spinel ferrite phase.

**Figure 2:**
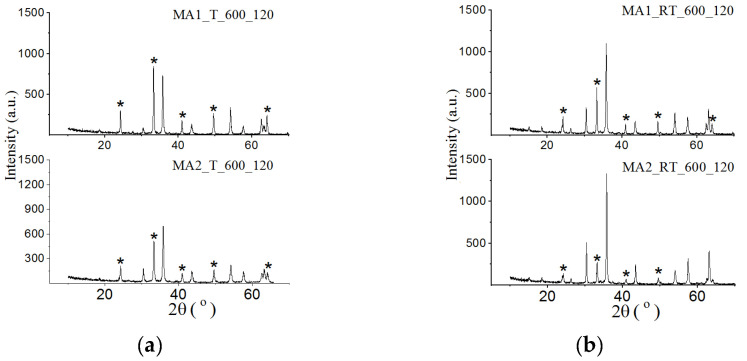
X-ray diffraction patterns of LiTi ferrite synthesized by T (**a**) and RT (**b**) heating at 600 °C for 120 min from Li_2_CO_3_/Fe_2_O_3_/TiO_2_ mixture mechanically activated at different energy intensity. *—Fe_2_O_3_.

Figure 2 shows the diffraction patterns of samples synthesized from mechanically activated reagents at 600 °C for 120 min. All diffraction patterns show reflections from the spinel phase and from the initial Fe_2_O_3_ reagent. At increased rotation speed during powder milling, the amount of the synthesized spinel phase increases with both types of heating, yet a large amount of unreacted iron oxide remains. A similar pattern was observed for all synthesis times.

**Figure 3:**
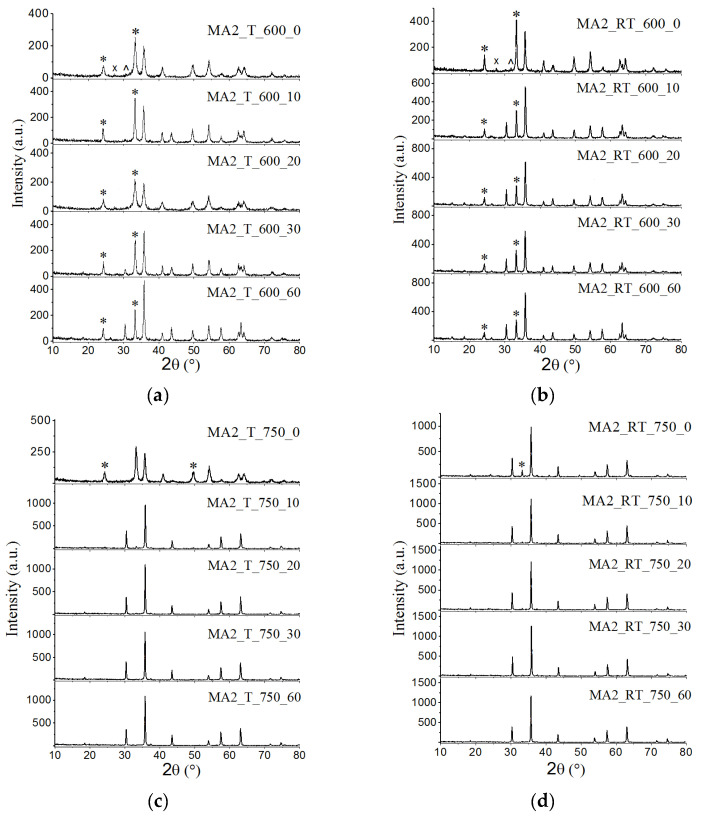
X-ray diffraction patterns of LiTi ferrite synthesized by T (**a**,**c**) and RT (**b**,**d**) heating at 600 (**a**,**b**) and 750 (**c**,**d**) °C for different isothermal holding times. *—Fe_2_O_3_, ^—Li_2_CO_3_, ^X^—TiO_2_.

As shown in Figure 3, as synthesis temperature increased to 750 °C, the amount of ferrite formed significantly increases. In this case, samples synthesized for 10 min or longer contain only the spinel phase with both types of heating. At first sight, a similar type of the XRD indicates the similarity in the phase composition of these samples. As will be shown below, in reality it was different. Table 3 and Table 4 present XRD and other data for the spinel phase synthesized for 120 min from mechanically activated reagents using different rotation speeds of cups.

**Figure 4:**
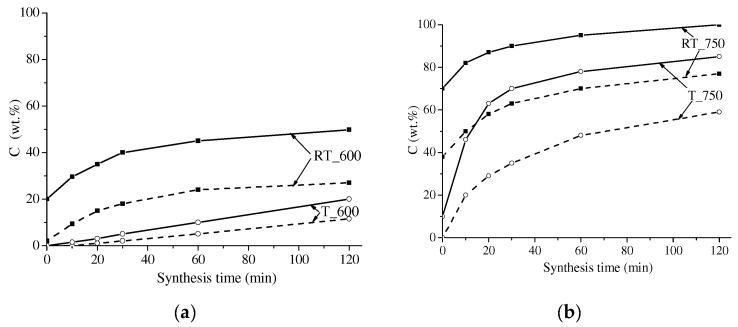
Concentration of spinel phases in LiTi ferrite synthesized at 600 (**a**) and 750 (**b**) °C: solid lines—compacted samples; dotted line—powdered samples.

**Figure 5:**
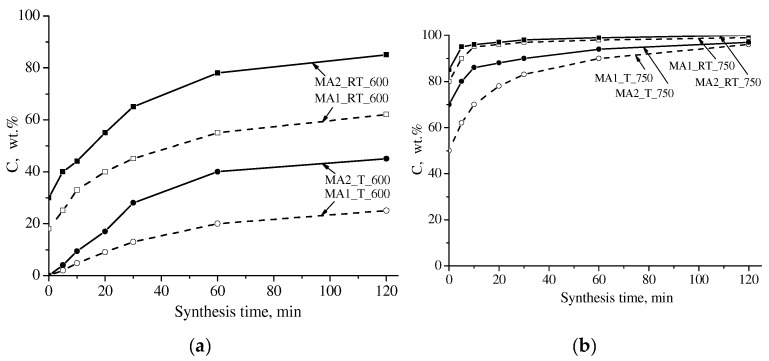
Concentration of spinel phases in LiTi ferrite synthesized at 600 (**a**) and 750 (**b**) °C from mechanically activated Li_2_CO_3_/Fe_2_O_3_/TiO_2_ reagents mixture: solid lines—activation at 2220 rpm; dotted line—activation at 1290 rpm.

Figure 4 and Figure 5 show XRD patterns of the dependences of the spinel phase concentration in the synthesized samples at the synthesis time. Under thermal heating, at a synthesis temperature of 600 °C, the content of the spinel ferrite phase in the samples slowly increased with increased synthesis time. In this case, the concentration curves are linear. In all other modes, the concentration curves indicate the region of rapid accumulation of the spinel phase; its time interval depends on the treatment mode and the type of sample preparation; further it showed a slow increase in concentration over time.

Under electron-beam heating of compacted samples at 750 °C, the area of fast accumulation of the spinel phase in the concentration curves was partially shifted to the nonisothermal heating stage; for the given synthesis temperature, it was about 5 min. This effect is more pronounced in mechanically activated samples heated at 750 °C. In the latter case, a large amount of the spinel phase formed at the heating stage could be observed in the samples synthesized using both types of heating.

The behavior of the curves for the ferrite phase formation allows the assumption that synthesis involves diffusion interaction between the initial reagents, which initiates the formation of transition phases. Then the transition phases and phases of the initial reagents interact with each other to form lithium-substituted ferrite phases, namely, lithium-titanium ferrite phases. This assumption was confirmed by the previously obtained results, which show the dynamics of changes in the formation of intermediate reaction products during synthesis of substituted lithium ferrites [43].

The results obtained in [43] showed that the synthesis of substituted lithium ferrites at the initial heating stage causes formation of unsubstituted lithium ferrite of the Li_0.5_Fe_2.5_O_4_ composition. Therefore, the concentration dependences obtained in this study for lithium-titanium ferrite are like those obtained for lithium ferrite in [43]. Considering that during heating of the Fe_2_O_3_/Li_2_CO_3_ mixture by a high-energy electron beam, the initial rate of lithium ferrite formation sharply increases, the initial stage of the RT synthesis of lithium-titanium ferrite involves the formation of unsubstituted lithium ferrite phases. It is also known [10] that stable spinel compounds, such as Li_2_TiO_3_, Li_4_Ti_5_O_12_, Li_4_Ti_5_O_12_ and Li_4_Ti_5_O_12_, can be formed during lithium-titanium ferrite synthesis [10]. Apparently, during synthesis of lithium-titanium ferrites from Fe_2_O_3_/Li_2_CO_3_/TiO_2_ powder reagents, similar lithium-titanium oxide compounds are also formed in parallel with lithium ferrite at the initial stages of lithium-titanium ferrite formation. At later synthesis stages, lithium-titanium ferrite is formed due to the interaction between the initially formed phases.

### 3.2. Study of Specific Saturation Magnetization

**Figure 6:**
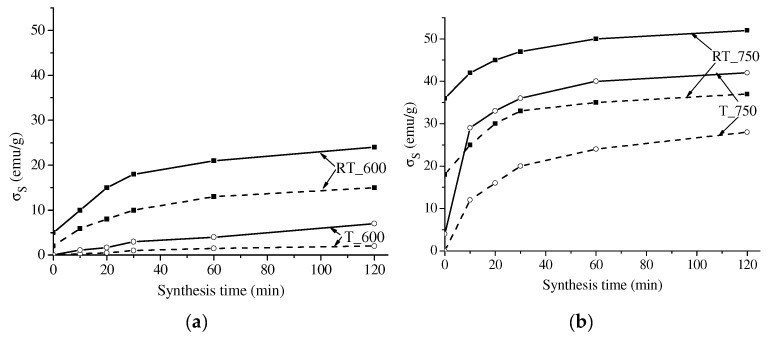
Specific saturation magnetization of LiTi ferrite as a function of synthesis time: solid lines—compacted samples; dotted line—powdered samples. Synthesis is at 600 (**a**) and 750 (**b**) °C.

**Figure 7:**
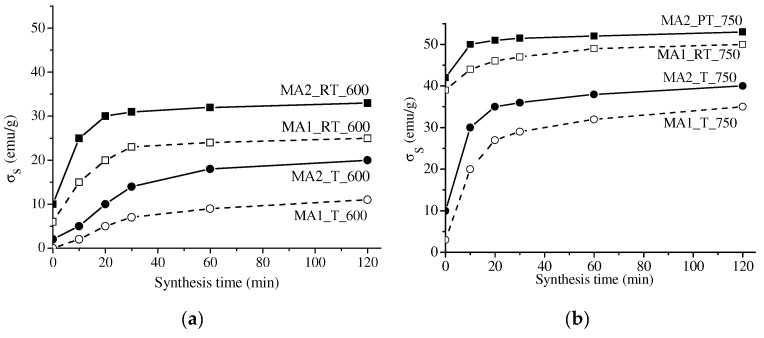
Specific saturation magnetization of LiTi ferrite as a function of synthesis time: solid lines—reagents activation at 2220 rpm; dotted line—reagents activation at 1290 rpm. Synthesis is at 600 (**a**) and 750 (**b**) °C.

Figure 6 and Figure 7 present the specific saturation magnetization of the samples versus their synthesis time. The value of σ_S_ for all the samples was observed to grow at increased synthesis time due to the increased magnetic phase. The behavior of the σ_S_ curves was generally like that of the above concentration curves. At a short synthesis time, the σ_S_ value sharply increased; at a synthesis time exceeding 30 min, the sample magnetization varied insignificantly.

The specific saturation magnetization of lithium-titanium ferrite is known to be lower than that of lithium ferrite. Therefore, the fluctuations in the behavior of the kinetic curves are related to competing processes in the formation of both unsubstituted lithium ferrite and substituted lithium-titanium ferrites.

Comparison of the curves revealed a higher degree of magnetization in the samples synthesized from mechanically activated reagents as compared to those obtained from powder or compacted samples. In addition, at increased grinding energy intensity, the content of the magnetic phase in the samples grew. In this case, the samples mechanically activated at 2220 rpm and synthesized by electron-beam heating show a high magnetization value of 50 emu/g, which is like the data reported in [24,45]. This is related to the decreased particle size and increased inter-particle contact area caused by mechanical treatment, which increases the rate of ferrite formation. 

### 3.3. Thermomagnetometric Analysis of Samples

A thermomagnetometric (TM) analysis was performed to estimate the Curie temperature of the synthesized samples. As reported in [47], the XRD and TM data can be used to estimate the complete phase composition of ferrites more accurately.

**Figure 8:**
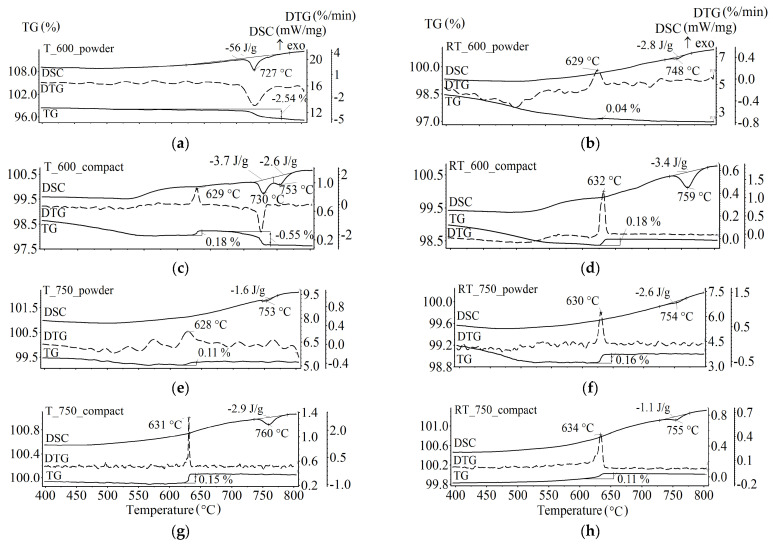
TG/DSC analysis of LiTi ferrite synthesized at 600 and 750 °C from powdered and compacted Li_2_CO_3_/Fe_2_O_3_/TiO_2_ reagents mixture. (**a**,**b**)—powdered samples synthesized at 600 °C by T and RT heating respectively; (**c**,**d**)—compacted samples synthesized at 600 °C by T and RT heating respectively; (**e**,**f**)—powdered samples synthesized at 750 °C by T and RT heating respectively; (**g**,**h**)—compacted samples synthesized at 600 °C by T and RT heating respectively.

**Figure 9:**
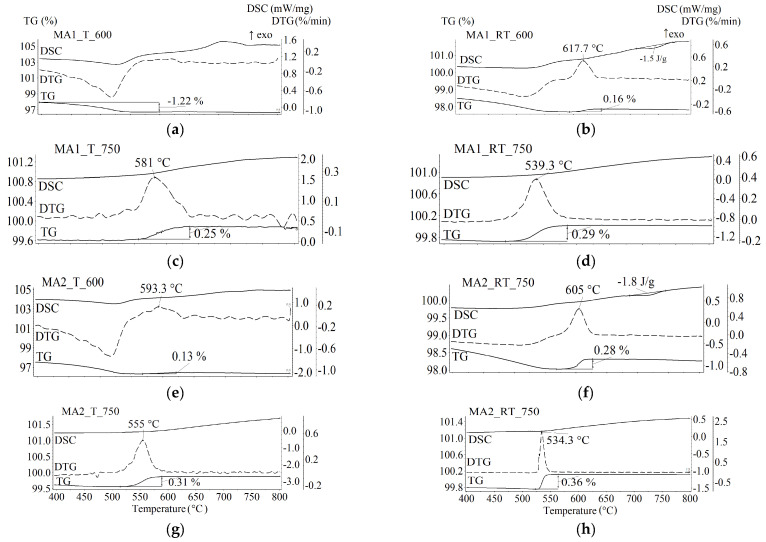
TG/DSC analysis of LiTi ferrite synthesized at 600 and 750 °C for 120 min from Li_2_CO_3_/Fe_2_O_3_/TiO_2_ mixture mechanically activated at different energy intensity. (**a**,**b**)—mechanically activated at rotation speed of 1290 rpm samples synthesized at 600 °C by T and RT heating respectively; (**c**,**d**)—mechanically activated at rotation speed of 1290 rpm samples synthesized at 750 °C by T and RT heating respectively; (**e**,**f**)—mechanically activated at rotation speed of 2220 rpm samples synthesized at 600 °C by T and RT heating respectively; (**g**,**h**)—mechanically activated at rotation speed of 2220 rpm samples synthesized at 750 °C by T and RT heating respectively.

Figure 8 and Figure 9 show the results of the TM analysis, including thermogravimetric (TG) data in the external magnetic field, derivative thermogravimetric (DTG) data, and differential scanning calorimetry (DSC) data for the samples synthesized for 120 min. The TG and DSC measurements were performed simultaneously at a high heating rate of 50 K/min.

For the powder sample synthesized at 600 °C by thermal heating (Figure 8, T_600_powder sample), the decreased weight in the TG curve and DSC peak at 727 °C is caused by the CO_2_ release due to the synthesis reaction between the residues of the Fe_2_O_3_/Li_2_CO_3_/TiO_2_ initial reagents according to Equation (1). The calculated weight loss corresponding to the complete reaction equals 6.17% with an enthalpy of 100 J/g [48]. Therefore, the obtained value of 2.54% with an enthalpy of 56 J/g in the TG curve corresponds to approximately 41% completion of the reaction. For the compact sample (Figure 8, T_600_compact), the observed reaction was associated with the presence of initial reagents that did not react during synthesis, but were less numerous. In this case, the peak observed in the DSC curve at 753 °C is due to the “order-disorder” phase transition in the synthesized lithium ferrite α-Li_0.5_Fe_2.5_O_4_. According to [22], the enthalpy of a complete transition is 12 J/g. Therefore, the concentration of the synthesized unsubstituted lithium ferrite estimated from the DSC peak is 21.6 wt.%, which is similar to the spinel phase concentration of 20 wt% according to the XRD analysis of this sample. The presence of Li_0.5_Fe_2.5_O_4_ in this sample is evidenced by the Curie temperature, which corresponds to the magnetic phase transition and equals 629 °C. At this temperature, the TG curve shows a weight jump caused by termination of the sample interaction with the external magnetic field. The obtained value of the Curie point is similar to that reported in [22,49].

Figure 8 shows that the height of the weight jump in the magnetic phase transition and the peak area of the DSC α→β transition in the Li_0.5_Fe_2.5_O_4_ phase, which characterize the concentration content of lithium ferrite, depend on the synthesis temperature, heating method, and sample activity. When the temperature of thermal synthesis was increased to 750 °C, the content of lithium ferrite increases, whereas the increased RT synthesis temperature reduces the content of the Li_0.5_Fe_2.5_O_4_ phase in the samples.

The samples preliminarily mechanically activated and then synthesized do not contain unsubstituted lithium ferrite (Figure 9). The Curie temperature depends on the rotation speed of mechanical grinding. Variation in the Curie temperature corresponds to the formation of lithium-titanium ferrites with different titanium substitutions [47]. Thus, the formation of lithium-titanium ferrite is more intensive during synthesis of samples from mechanically activated powders. In this case, samples mechanically activated at 2220 rpm and synthesized by electron-beam heating show a Curie temperature of 534 °C, which is like that for the T_C_ of Li_0.6_Fe_2.2_Ti_0.2_O_4_ ferrite [47].

Apparently, mechanical grinding of the mixture of different powders not only decreases the particle size, but also significantly increases the number of triple contacts Li_2_CO_3_—Fe_2_O_3—_TiO_2_, which stimulates a rapid formation of substituted lithium-titanium ferrites.

Electron-beam heating of the studied material yields many free electrons, which can briefly reduce the charge of cations (Fe^3+^ и Ti^4+^). The change in the charge of cations decreases the energy of their electrostatic interaction with surrounding anions, thereby increasing their mobility. Consequently, an accelerated fusion reaction under RT heating can occur only if the initial stage of solid-phase interaction is limited by the diffusion of multiply charged Fe^3+^ and Ti^4+^ cations. In addition, local overheating of interfaces can occur due to nonradiative recombination of radiation-generated electronic excitations at the interfaces. In mechanically activated powders, the density of inter-particle contacts increases, which leads to local temperature gradients during RT treatment and changes the diffusion activity of particles.

The increased time of electron-beam heating minimizes the contribution of the radiation component to the diffusion driving force. This is due to the slower interaction of transition phases and subsequent formation of single-phase lithium-titanium ferrite.

**Figure 10:**
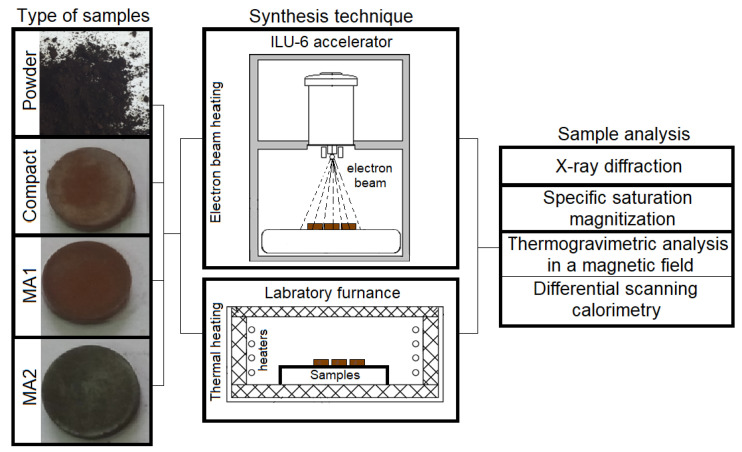
Technological scheme of lithium-titanium ferrite synthesis.

Based on the obtained results, technological regimes were developed for efficient synthesis of lithium-titanium ferrites. Figure 10 presents the scheme that includes the mechanical activation of mixtures of ferrite reagents Li_2_CO_3_—Fe_2_O_3_—TiO_2_ and their subsequent heating by a pulsed electron beam.

## 4. Conclusions

The XRD and TM analyses were employed to study the solid-phase synthesis of lithium-titanium ferrite with the chemical composition of Li_0.6_Fe_2.2_Ti_0.2_O_4_ under electron-beam heating of a mixture of Fe_2_O_3_—Li_2_CO_3_—TiO_2_ initial reagents at 600 and 750 °C, and to analyze the specific saturation magnetization. The initial reagents were used in the form of a powder, a compact, and a powder mechanically activated at different speeds. The parameters of the samples synthesized by electron-beam heating were compared with those prepared using the conventional ceramic technology.

It was shown that the rate of lithium-titanium ferrite synthesis depends on the history of powder reagents, type of heating, and temperature and time of synthesis. Increased temperature and time of synthesis increased the concentration of the spinel ferrite phase in the samples, which is typical of ferrite synthesis. The compaction of samples increased the rate of ferrite synthesis, but the temperature-time regimes used caused the synthesis of unsubstituted lithium ferrite of Li_0.5_Fe_2.5_O_4_, which is a transition product of the reaction during synthesis of substituted lithium ferrites. The formation of lithium ferrite was confirmed by the registered Curie temperature, equal to 631–634 °C depending on the heating method.

Preliminary mechanical activation of the Fe_2_O_3_—Li_2_CO_3_—TiO_2_ initial powder reagents significantly accelerated ferrite formation under both types of heating. When the energy intensity of grinding that depends on the cup rotation speed (1290 and 2220 rpm) grew, the concentration of the synthesized spinel phase increased, which is associated with the formation of lithium substituted phases of variable composition.

Under electron-beam heating mixtures of initial reagents, the rate of lithium-titanium ferrite formation was much higher than that during conventional thermal synthesis. This was evidenced by a faster transformation of the initial reagents into spinel ferrite phases in the entire temperature range. In this case, the samples mechanically activated at a high rotation speed of 2220 rpm and synthesized by electron-beam heating at 750 °C were characterized by a single-phase ferrite composition of Li_0.6_Fe_2.2_Ti_0.2_O_4_, which was confirmed by data on the Curie temperature (534 °C) and specific saturation magnetization (50 emu/g).

The paper proposes a technological scheme for efficient synthesis of substituted lithium-titanium ferrites at a lower temperature (750 °C) and a shorter synthesis time (120 min) compared to traditional ceramic technology, which requires the use of high synthesis temperature of about 1000 °C and long synthesis time over 10 h.

## Figures and Tables

**Table 1:** Structural and magnetic properties of LiTi ferrite synthesized from powdered and compacted Li_2_CO_3_/Fe_2_O_3_/TiO_2_ mixture.

Sample	C_spinel_ (wt.%)	Lattice Parameter (Ǻ)	Crystallite Size (nm)	Microstrain Δd/d·10^3^	T_c_ (°C)	σ_S_ (emu/g)
T_600_120 powder	11.5	8.325	53	0.6	-	1.3
T_600_120 compact	20.0	8.330	77	0.6	629	6.6
RT_600_120 powder	28.4	8.327	70	0.4	629	14.9
RT_600_120 compact	49.8	8.329	88	0.7	632	24.0

**Table 2:** Structural and magnetic properties of LiTi ferrite synthesized from powdered and compacted Li_2_CO_3_/Fe_2_O_3_/TiO_2_ mixture.

Sample	C_spinel_ (wt.%)	Lattice Parameter (Ǻ)	Crystallite Size (nm)	Microstrain Δd/d·10^3^	T_c_ (°C)	σ_S_ (emu/g)
T_750_120 powder	59.0	8.327	120	0.5	628	27.9
T_750_120 compact	83.0	8.331	130	1.1	631	42.0
RT_750_120 powder	77.0	8.330	141	0.3	630	37.6
RT_750_120 compact	100	8.334	127	1.1	634	51.5

**Table 3:** Structural and magnetic properties of LiTi ferrite synthesized from mechanically activated Li_2_CO_3_/Fe_2_O_3_/TiO_2_ mixture.

Sample	C_spinel_ (wt.%)	Lattice Parameter (Ǻ)	Crystallite Size (nm)	Microstrain Δ*d/d*·10^3^	T_c_ (°C)	σ_S_ (emu/g)
MA1_T_600	18.9	8.321	45	1.0	-	8.1
MA1_T_750	98.0	8.337	84	0.8	581	34.0
MA1_RT_600	54.5	8.328	88	0.9	618	27.0
MA1_RT_750	100	8.338	142	0.6	539	49.7

**Table 4:** Structural and magnetic properties of LiTi ferrite synthesized from mechanically activated Li_2_CO_3_/Fe_2_O_3_/TiO_2_ mixture.

Sample	C_spinel_ (wt.%)	Lattice Parameter (Ǻ)	Crystallite Size (nm)	Microstrain Δ*d/d*·10^3^	T_c_ (°C)	σ_S_ (emu/g)
MA2_T_600	37.1	8.322	42	1.0	593	18.3
MA2_T_750	99.0	8.335	80	0.6	555	38.5
MA2_RT_600	78.9	8.333	63	0.4	605	32.1
MA2_RT_750	100	8.337	49	0.8	534	51.0

## Data Availability

The data presented in this study are available from the corresponding authors upon reasonable request.
